# Anti-tumor efficacy and biomarker evaluation of agonistic anti-OX40 antibodies in preclinical models

**DOI:** 10.1186/2051-1426-2-S3-P105

**Published:** 2014-11-06

**Authors:** Mahrukh Huseni, Klara Totpal, Changchun Du, Katie Dalpozzo, Jing Zhu, Deepali Rishipathak, Erin McNamara, Bernadette Jonshtone, Priti S Hegde, Ina Rhee, Lisa Damico-Beyer, Jeong Kim

**Affiliations:** 1 Genentech, Inc., San Francisco, CA, USA

## Background

OX40, a TNFRSF member, is a co-stimulatory molecule expressed on antigen experienced effector T (Teff) and regulatory T (Treg) cells, including infiltrating cells in mouse and human tumors. Activation of OX40 by agonistic antibodies is hypothesized to promote anti-tumor immunity by enhancing Teff activation and inhibiting Treg mediated suppression. We conducted *in vitro *and *in vivo *preclinical studies to characterize co-stimulatory activity, evaluate anti-tumor efficacy and identify predictive and pharmacodynamic (PD) biomarkers of agonistic anti-OX40 antibodies.

## Methods

*In vitro *evaluation of Teff costimulation and Treg inhibition was conducted with MOXR0916, a humanized anti-human OX40 mAb, using purified T cells from healthy human donors. *In vivo *anti-tumor efficacy was studied using PRO307205, a murine anti-mouse Ab, in multiple established syngeneic mouse tumor models. Baseline tumor immune environment was characterized using multiplex gene expression analysis of 93 immune related genes. Circulating and tumor-based PD biomarkers of PRO307205 activity were evaluated in EMT6 and JC tumor models using flow cytometry and multiplex gene expression assays.

## Results

Engagement of OX40 by MOXR0916 in the presence of anti-CD3 stimulation enhanced IFN-γ production and proliferation of Teff cells, and suppressed Treg cell function in naïve T cell co-cultures. Administration of PRO307205 resulted in a spectrum of anti-tumor activity *in vivo*, including durable tumor regression in the EMT6 model and modest tumor growth inhibition in the JC model. Higher baseline expression of immune activation markers and lower expression of immune inhibitory and Th2 associated markers were predictive of better anti- tumor response to PRO307205. Biomarkers of PD activity in EMT6 and JC models included reduction of Treg cells in blood and tumors and augmentation of cytotoxic immune function in tumors as evidenced by increase in gene expression of IFN-γ, granzymes, and perforin. PD modulation that corresponded with differential anti-tumor activity included sustained enhancement of CD8 T cell infiltration and expression of co-stimulatory and memory T cell markers such as CD86, ICOS, CXCR3, and IL7R in EMT6 tumors but not in JC tumors.

## Conclusions

Our findings show that agonistic anti-OX40 Abs induce potent T cell activation and promote anti-tumor immunity and efficacy in preclinical systems. Biomarkers identified in these studies may be utilized to validate mechanism of action, inform dose finding, and guide patient selection in MOXR0916 clinical trials.

